# Volume Load-Induced Right Ventricular Failure in Rats Is Not Associated With Myocardial Fibrosis

**DOI:** 10.3389/fphys.2021.557514

**Published:** 2021-02-26

**Authors:** Quint A. J. Hagdorn, Kondababu Kurakula, Anne-Marie C. Koop, Guido P. L. Bossers, Emmanouil Mavrogiannis, Tom van Leusden, Diederik E. van der Feen, Rudolf A. de Boer, Marie-José T. H. Goumans, Rolf M. F. Berger

**Affiliations:** ^1^Center for Congenital Heart Diseases, Department of Pediatric Cardiology, Beatrix Children’s Hospital, University Medical Center Groningen, University of Groningen, Groningen, Netherlands; ^2^Department of Cell and Chemical Biology, Leiden University Medical Center, Leiden, Netherlands; ^3^Department of Cardiology, University Medical Center Groningen, University of Groningen, Groningen, Netherlands

**Keywords:** right ventricular remodeling, right ventricular remodeling and fibrosis, right ventricular failure, fibrosis, volume load, aortocaval shunt, tetralogy of Fallot

## Abstract

**Background:**

Right ventricular (RV) function and failure are key determinants of morbidity and mortality in various cardiovascular diseases. Myocardial fibrosis is regarded as a contributing factor to heart failure, but its importance in RV failure has been challenged. This study aims to assess whether myocardial fibrosis drives the transition from compensated to decompensated volume load-induced RV dysfunction.

**Methods:**

Wistar rats were subjected to aorto-caval shunt (ACS, *n* = 23) or sham (control, *n* = 15) surgery, and sacrificed after 1 month, 3 months, or 6 months. Echocardiography, RV pressure-volume analysis, assessment of gene expression and cardiac histology were performed.

**Results:**

At 6 months, 6/8 ACS-rats (75%) showed clinical signs of RV failure (pleural effusion, ascites and/or liver edema), whereas at 1 month and 3 months, no signs of RV failure had developed yet. Cardiac output has increased two- to threefold and biventricular dilatation occurred, while LV ejection fraction gradually decreased. At 1 month and 3 months, RV end-systolic elastance (Ees) remained unaltered, but at 6 months, RV Ees had decreased substantially. In the RV, no oxidative stress, inflammation, pro-fibrotic signaling (TGFβ1 and pSMAD2/3), or fibrosis were present at any time point.

**Conclusions:**

In the ACS rat model, long-term volume load was initially well tolerated at 1 month and 3 months, but induced overt clinical signs of end-stage RV failure at 6 months. However, no myocardial fibrosis or increased pro-fibrotic signaling had developed. These findings indicate that myocardial fibrosis is not involved in the transition from compensated to decompensated RV dysfunction in this model.

## Introduction

Right ventricular (RV) function and failure are key determinants of morbidity and mortality in various cardiovascular diseases. When exposed to chronic abnormal loading conditions, as is often the case in patients with congenital heart disease, deterioration of RV function and progression toward failure can occur (Friedberg and Reddy, 2019). Abnormal loading conditions consist either of increased pressure load, for example in the case of pulmonary hypertension (PH), or increased volume load, for example, in the case of pulmonary regurgitation in repaired tetralogy of Fallot (rTOF). Both types of ventricular loading induce very distinct phenotypes (Toischer et al., 2010; Bartelds et al., 2011; Borgdorff M. A. J. et al., 2013). In PH, increased RV pressure load leads to rapid RV deterioration and failure (Zijlstra et al., 2014), whereas RV volume loading in patients with rTOF is generally tolerated for decades until eventually failure occurs (Kuehne et al., 2003; Bouzas et al., 2005). Also experimentally, RV pressure load demonstrates an unfavorable clinical course, compared to volume load, even when the duration of adverse loading and mean total wall stress are similar (Toischer et al., 2010; Borgdorff M. A. J. et al., 2013). Therefore, to induce volume load-associated RV failure, long-term animal models are required. RV pressure load has been studied rather extensively, compared to RV volume load (Friedberg and Redington, 2014; Iacobazzi et al., 2016; Bossers et al., 2018; Lahm et al., 2018). However, knowledge obtained from the pressure-loaded RV cannot be transferred one-by-one to our understanding of RV volume load. Yet, the distinct phenotype of volume load-induced RV failure is seriously understudied, while the group of patients with rTOF at risk of volume-load induced RV failure is growing (Bouzas et al., 2005; Bossers et al., 2018).

Fibrosis is a process of excessive deposition of extracellular matrix that is observed in various types of heart failure, and is generally regarded as a detrimental factor and a potential treatment target in heart failure therapy (Suthahar et al., 2017; de Boer et al., 2019). Indeed, RV adaptation to pressure load has been shown to be associated with progressive myocardial fibrosis. However, the causal relation between fibrosis and failure has recently been challenged by the lack of beneficial effects of anti-fibrotic therapies on RV performance (Borgdorff M. A. J. et al., 2013; Crnkovic et al., 2018; Andersen et al., 2019). Also, animals with symptomatic RV failure were shown to actually have less RV fibrosis, compared to animals without clinical symptoms (Borgdorff et al., 2015). In the volume-loaded RV, the role of myocardial fibrosis and its relation with ventricular failure is even more unclear (Bossers et al., 2018). In patients with rTOF, often exposed to RV volume loading, indirect imaging markers have suggested both focal and interstitial fibrosis, correlated to function, dilatation and the degree of volume load (Haggerty et al., 2017; Hanneman et al., 2017; Yim et al., 2017; Cochet et al., 2019). However, since these studies only report associations, it remains unclear whether this process plays either a causal and detrimental, or secondary and reactive role in RV remodeling to volume loading. Furthermore, these studied patients all have underwent at least one cardiac surgery at young age with accompanying scarring and cross-clamp time, and were subjected to pressure load pre-operatively. These are confounding factors when studying fibrosis. Distinguishing the effects of volume overload from these confounders requires animal studies, but experimental studies on chronic RV volume loading are scarce. Therefore, to assess whether myocardial fibrosis drives the transition from compensated to decompensated volume load-induced RV dysfunction, this study aimed to describe the temporal pattern of ventricular adaptation, fibrosis and pro-fibrotic signaling in a long-term model of volume load induced RV failure.

## Materials and Methods

### Animal Experiments

Thirty-nine male Wistar WU rats (160–180 g, Charles River) were randomly subjected to aorto-caval shunt surgery (ACS, *n* = 24) or sham surgery (control, *n* = 15). The ACS was performed as described previously (van der Feen et al., 2017). In short, rats were anesthetized by inhalation of 2–3% isoflurane, and were given buprenorphine 0.01 mg/kg subcutaneously for post-operative analgesia. After mid-line laparotomy, a puncture was made with a 18G needle through the abdominal aorta toward the inferior caval vein. The puncture site in the aorta was closed with tissue glue. Sham surgery consisted of laparotomy and application of tissue glue on the abdominal aorta. One rat died perioperatively and one ACS rat was excluded, as assessment of shunt during termination revealed no shunting. All animal experimental protocols have been approved by the Dutch Central Ethical Committee for Animal Experiments and the Animal Care Committee of the University Medical Center Groningen (permit number: AVD105002015134). The experiments were conducted according to the guidelines from Directive 2010/63/EU of the European Parliament on the protection of animals used for scientific purposes and the ethical standards laid down in the 1964 Declaration of Helsinki and its later amendments.

Animals were sacrificed at three different time points: 1 month after surgery (ACS *n* = 7, control *n* = 3), 3 months after surgery (ACS *n* = 8, control *n* = 6), and 6 months (6 m) after surgery (ACS *n* = 8, control *n* = 6). Prior to termination, echocardiography and invasive RV pressure–volume analysis was performed, as described below.

### Echocardiography

Echocardiographic assessment was performed using a Vivid Dimension 7 and 10S-transducer (GE Healthcare, Waukesha, WI, United States) under inhalation of 2–3% isoflurane. Flow-measurements of the aorta and pulmonary artery were performed, and LV cardiac output (CO) and pulmonary artery acceleration time (PAAT) were calculated. In the apical four-chamber view, LV internal diastolic diameter (LVIDd), RV internal diastolic diameter (RVIDd), tricuspid annular plane systolic excursion (TAPSE, using M-mode), and the presence of regurgitation of the mitral valve or tricuspid valve were assessed. LV ejection fraction (EF) was assessed using M-mode in the short axis, using Teichholz’s formula. RVIDd, LVIDd, and TAPSE were indexed by dividing by tibia length (TL), and LV CO was indexed by dividing by tibia length to the power of three (TL^3^), as described previously (Hagdorn et al., 2019).

### Invasive Hemodynamics

Invasive RV pressure-volume analysis was performed while intubated and ventilated with 2–3% isoflurane in oxygen as described previously (Borgdorff M. A. J. et al., 2013). First, a two-lead electrocardiogram (ECG) was made for rhythm analysis and QRS duration measurement. After thoracotomy, the catheter (pressure-admittance, 1.9F, 6 mm spacing, Transonic, Ithaca, NY, United States) was introduced through the apex toward the RV outflow tract. Analyses were performed using Circlab 2015, version 10.8 (P. Steendijk, Leiden University Medical Center, Leiden, Netherlands). Echocardiographic stroke volume was used to calibrate volumetric measurements. The maximal rate of pressure increase (dP/dt_max_) and pressure decrease (dP/dt_min_) was calculated and divided by maximal pressure (P_max_). End-diastolic pressure (EDP) and arterial elastance (Ea) were obtained from single-beats in steady state. After gradually decreasing preload by slow constriction of the inferior caval vein, end-systolic and end-diastolic elastance (Ees and Eed, respectively) were calculated by measuring the slope of the end-systolic and end-diastolic points of selected heartbeats.

### Termination

All animals were euthanized by exsanguination and extraction of the heart under inhalation of 2–3% isoflurane. The presence of pleural effusion, ascites, or visible liver edema/cirrhosis was registered. Cardiac weights (LV, RV, both atria) and TL were measured. Cardiac weights were indexed as described previously (Hagdorn et al., 2019). The heart was partially snap-frozen for gene and protein analyses, and partially formalin fixed and embedded in paraffin for histological analyses.

### Histology

Cardiomyocyte cross-sectional area (CCSA) was measured on wheat germ agglutinin (WGA) stained paraffin sections. Capillary/myocyte ratio was measured by dividing the counted amount of capillaries on five views of 100 × 100 μm by the expected amount of cardiomyocytes (calculated as area of analysis divided by mean CSSA). For assessment of the amount of fibrosis, Masson’s trichrome staining (all time points) and Sirius red staining (as confirmation, only 6 months time point) was performed on paraffin whole sections of the heart, and analyzed using Image Scope 11 (Aperio Technologies, Vista, CA). The amount of fibrosis was quantified, per ventricle, as the blue-stained (Masson’s trichrome) or red-stained (Sirius red) percentage of the total tissue area, excluding major vessels. Pulmonary vascular histology was qualitatively assessed on Verhoeff stained paraffin sections of the left lung, based on previous experience (van der Feen et al., 2017). Macrophage infiltration was assessed by means of CD69 immunohistochemistry staining.

### Gene Expression

RNA was isolated from snap-frozen tissue using Trizol reagent (Invitrogen) according to the manufacturer’s instructions and as described previously (Kurakula et al., 2015). cDNA was made using the iScript cDNA Synthesis Kit (Bio-Rad). Real-time reverse transcription PCR was performed using the MyIQ system (Bio-Rad). As an internal control for cDNA content of the samples, GAPDH was measured. Primer sequences are available on request. The relative gene expression was calculated using the 2^–Δ^ ^Δ^ ^*ct*^ method.

### Protein Levels

The amount of cardiac ATP was assessed using ATP Assay Kit (Abcam, ab83355) following manufacturer’s instructions. Using colorimetric quantification, levels of ATP were determined in both ACS (*n* = 5 per time point) and controls (*n* = 3 per time point) and normalized for protein concentration in each sample.

Whole-tissue lysates were prepared with NP-40 lysis buffer containing phosphatase and protease inhibitors (Sigma-Aldrich), and western blotting was performed as described previously (Kurakula et al., 2011, 2019). Equal amounts of protein was resolved by SDS-PAGE, and transferred to polyvinylidene difluoride (PVDF) membranes (Millipore). Blots were blocked with 5% non-fat dry milk in PBS/TBS with 0.1% Tween 20 (PBST/TBST) and incubated with primary antibodies overnight at 4°C. After washing with TBST buffer, membranes were incubated with appropriate horseradish peroxidase (HRP)-conjugated anti-mouse or anti-rabbit (GE Healthcare) secondary antibodies for 1 h in 5% non-fat milk. Antibodies were revealed using an ECL system (Fisher Scientific) and labeled proteins were detected with the imaging Chemidoc MP system (Bio-Rad). Protein expression was quantified using ImageJ software and normalized to vinculin as previously described (Kurakula et al., 2019). Antibodies directed against pSMAD2/3 (pS2 antibody; Persson et al., 1998), phosphorylated extracellular-regulated kinase 1/2 (pERK1/2; Cell signaling; #9101), Thr17 phosphorylated phospholamban (pPLN, Badrilla A010-13), total phospholamban (PLN, Thermo fisher MQ3-922), and vinculin (H300; Santa Cruz; sc-5573) were used.

### Statistics

Statistical analyses were performed using GraphPad Prism 7.02. Since groups are too small to test whether the distribution of variables is normal, data are displayed as median ± interquartile range. Most variables are expressed as relative to control. In these cases, fold-change compared to the time point-matched control group was assessed, where after the control groups were pooled. In variables where using a relative unit was not preferable, control groups were compared. If no significant differences in time were observed, control groups were pooled. Comparisons between ACS and control, and between time points for ACS were performed using Kruskal–Wallis test. False discovery rate was controlled for by means of the two-stage linear step-up procedure of Benjamini, Krieger, and Yekutieli as *post hoc* correction. Linear regression was used to assess the correlation between gene expression of transforming growth factor beta (TGFβ) 1 and bone morphogenetic protein receptor 2 (BMPR2). A P value below 0.05 was considered as statistically significant.

## Results

### Clinical State and Electrocardiography

**FIGURE 1:**
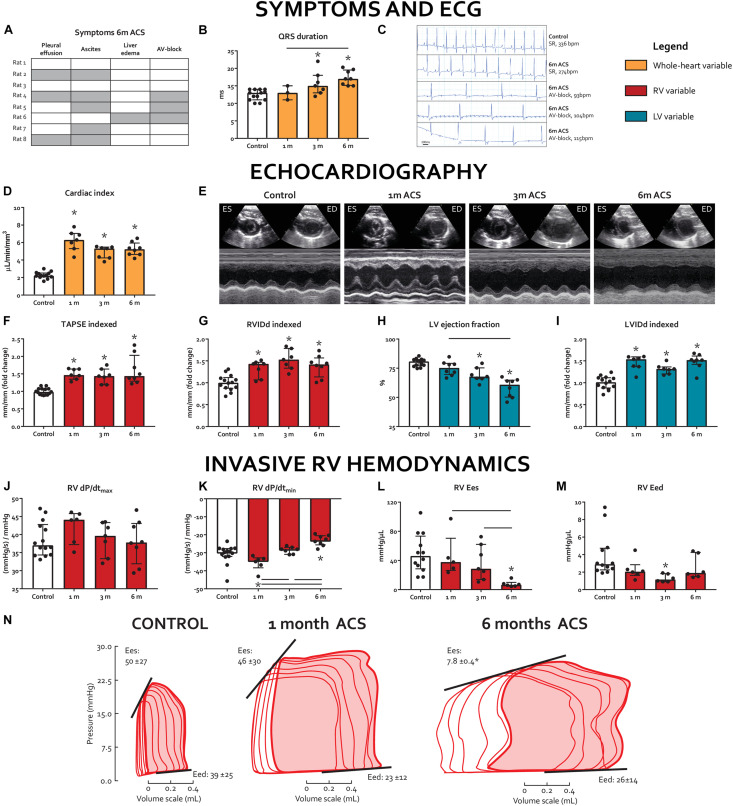
Long-term volume loading induces clinical signs of RV failure and biventricular dysfunction. **(A)** Prevalence of clinical signs of heart failure at 6 m, rows represent individual rats, gray cells indicate the presence of a symptom, and clear cells indicate the absence of a symptom. **(B)** QRS duration on ECG. **(C)** ECG registrations prior to termination. Top two are representative examples of sinus rhythm (SR) in a control rat and a 6-m ACS rat, bottom three are the rats that demonstrated AV-block. **(D-I)** Summary data and representative examples (short-axis M-mode through LV) of echocardiographic examinations. **(J-N)** Summary data and representative examples of invasive pressure-volume analyses. * represents significantly different from control, and bar represents significantly different between ACS groups.

Although initially well tolerated at 1 and 3 m after surgery without clinical symptoms, 6 out of 8 rats demonstrated signs of severe clinical heart failure 6 months after ACS (pleural effusion 3/8, ascites 5/8, and visible liver edema/cirrhosis 1/8, Figure 1A). On ECG, QRS duration increased in the 3 and 6 months ACS groups, compared to 1 month ACS and compared to control (Figure 1B). Notably, at 6 months after ACS, 3 out of 8 rats demonstrated a complete atrioventricular (AV) block, indicative of severe heart failure (Figure 1C). This AV block occurred immediately after intubation, and spontaneously converted into sinus rhythm in 2 out of 3 cases. Due to bradycardia in the rat with persisting AV block, PV-analyses of this rat were excluded from this study.

### Echocardiography

Echocardiography demonstrated that ACS induced substantial volume overload, as cardiac index in ACS rats increased more than three-fold at 1 month, compared to control, and remained significantly higher until 6 months (Figure 1D). RV internal diastolic diameter (RVIDd) and LV internal diastolic diameter (LVIDd) were significantly increased at all time points, compared to control (Figures 1G–I). Tricuspid annular plane systolic excursion (TAPSE), a volume-dependent measure of RV shortening, was increased in the ACS rats and remained so at all time points (Figure 1F). LV ejection fraction (EF) gradually decreased (Figures 1E,H).

### Invasive RV Hemodynamics

Results of invasive hemodynamics are displayed in Figures 1J–N. At 6 months, RV end-systolic elastance (Ees) was significantly decreased in the ACS group, compared to control, whereas RV dP/dt_max_ did not change in any of the ACS time points, compared to control. RV dP/dt_min_ was decreased in the 6 months ACS group, compared to control, which appeared to be progressive when compared to 1 month and 3 months ACS. RV maximal pressure (P_max_), RV end-diastolic pressure (EDP), and RV end-diastolic elastance (Eed) were not altered at any of the time points. Finally, RV Ees/arterial elastance (Ea) was significantly decreased after 6 months ACS. Heart rate was similar in ACS groups, compared to control.

### Remodeling and Stress

**FIGURE 2:**
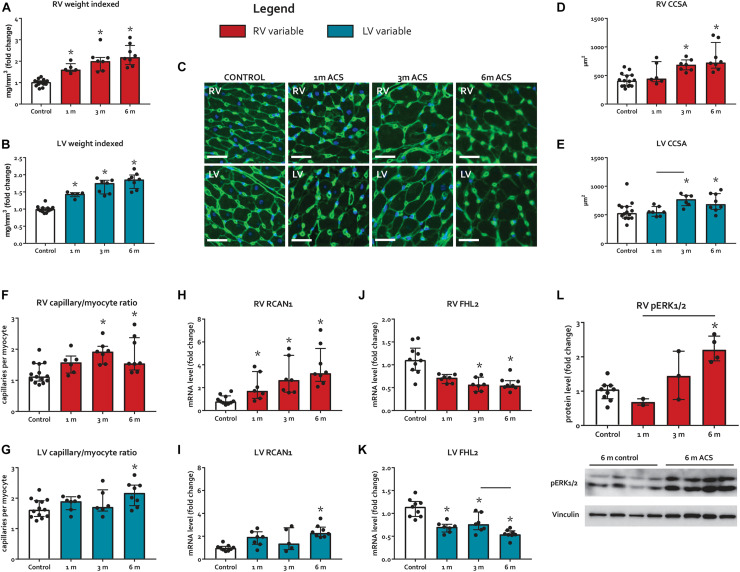
Myocardial remodeling and hypertrophy markers. **(A–B)** Ventricular weights, indexed for tibia length. **(C)** Representative examples of WGA-staining, on which cardiomyocyte cross-sectional area (CCSA) and capillary/myocyte ratio were determined. White bars represent 25 μm. **(D–G)** Summary data of CCSA and capillary/myocyte ratio. **(H–K)** mRNA levels of RCAN1 and FHL2. **(L)** Protein levels of RV pERK1/2. * represents significantly different from control, and bar represents significantly different between ACS groups.

ACS rats demonstrated progressive hypertrophy of both ventricles (Figures 2A,B) and atria (not displayed), compared to control. Cardiomyocyte cross-sectional area (CCSA) increased progressively in both ventricles, compared to control (Figures 2C–E). Capillary/myocyte ratio increased at 3 and 6 months in the RV and only at 6 months in the LV (Figures 2C,F,G). No increased macrophage infiltration was observed in ACS rats, compared to controls. Regulator of calcineurin 1 (RCAN1), an activator of pathological hypertrophy (Wilkins et al., 2004), increased in the RV at all time points, but only at 6 months in the LV of ACS rats when compared to control (Figures 2H,I). Gene expression of Four and a half LIM domains protein 2 (FHL2), a cardioprotective regulator of cardiac hypertrophy (Tran et al., 2016), decreased in in the RV at 3 and 6 months and in the LV at all time points, compared to control (Figures 2J,K). Protein levels of phosphorylated extracellular-regulated kinase (pERK)1/2, an hypertrophy regulator between eccentric and concentric growth (Kehat et al., 2011), increased in the RV at 6 months ACS, but were unchanged in the LV (Figure 2L).

**FIGURE 3:**
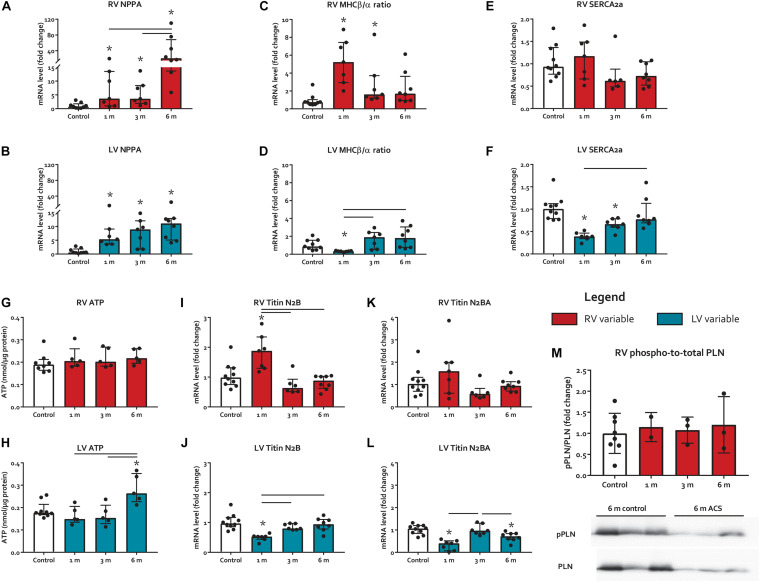
Myocardial stress and heart failure markers. **(A–F)** mRNA levels of myocardial stress and heart failure markers NPPA, MHCβ/α ratio, and SERCA2a. **(G-H)** Protein levels of ATP. **(I-L)** mRNA levels of titin isoforms N2B and N2BA. **(M)** phospho-to-total PLN mRNA level ratios. * represents significantly different from control, and bar represents significantly different between ACS groups.

Myocardial stress marker natriuretic peptide precursor A (NPPA) increased in both ventricles at all time points (Figures 3A,B). However, at 6 months, the level of NPPA increase in the RV was much higher than the LV (21-fold vs. 11-fold, respectively). The ratio of slow-twitch myosin heavy chain (MHC)β and fast-twitch MHCα, marker of switch to the fetal gene program due to cardiac injury (Mercadier et al., 1981), increased early in the RV at 1 month, and subsequently decreased over time (Figure 3C). In contrast, MHCβ/α ratio decreased in the LV at 1 month only (Figure 3D). Sarcoplasmic Reticulum Ca^2+^ ATPase 2a (SERCA2a), a regulator of intracellular Ca^2+^ that modulates excitation–contraction coupling (Frank et al., 2003), remained unaltered in the RV of ACS rats, but decreased in the LV at 1 month and 3 months ACS, compared to control (Figures 3E,F). Phospho-to-total phospholamban (pPLN/PLN) protein level ratios, a key regulator of SERCA2a (Frank et al., 2003), remained unaltered in both ventricles of ACS rats, compared to control (Figure 3M). Levels of ATP were unaltered in the RV in all time points, but were elevated in the LV at 6 months, compared to control (Figures 3G,H). Gene expression of titin’s stiffer isoform N2B increased at 1 month ACS in the RV, but decreased at 1 month ACS in the LV, compared to control (Figures 3I,J). Titin’s more compliant isoform N2BA remained unaltered in the RV, but was decreased at 1 month and 6 months ACS in the LV, compared to control (Figures 3K,L). The N2B/N2BA expression ratio was unaltered in both ventricles at all time points (not displayed). In accordance with the unaltered RV P_max_ in ACS rats, qualitative assessment of pulmonary histology demonstrated no signs of pulmonary vascular remodeling in ACS rats.

### Fibrosis and Pro-Fibrotic Signaling

**FIGURE 4:**
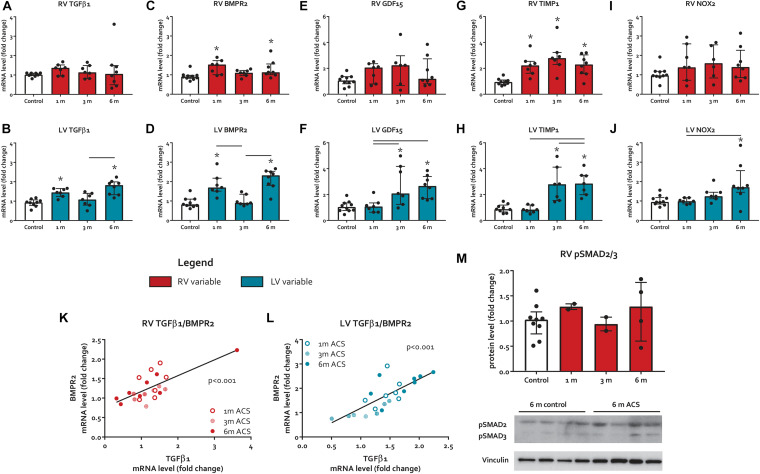
Pro-fibrotic signaling. **(A–J)** mRNA levels of pro-fibrotic signaling markers. **(K,L)** Scatter plot with linear regression line depicting the relation of TGFβ1 and BMPR2 for both ventricles. **(M)** RV protein levels of pSMAD2/3. * represents significantly different from control, and bar represents significantly different between ACS groups.

**FIGURE 5:**
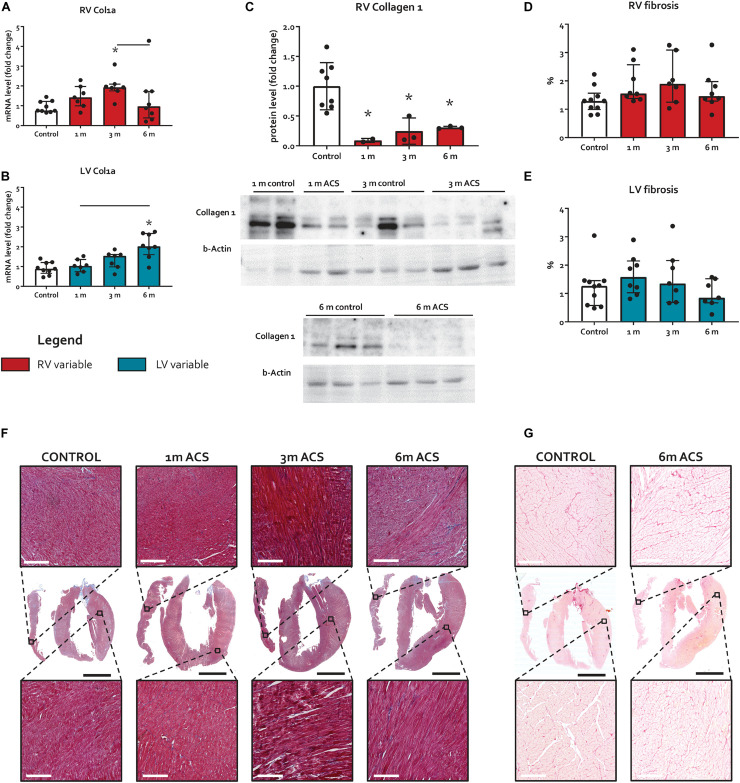
Long-term volume loading does not induce myocardial fibrosis. **(A,B)** mRNA levels of Col1a for both ventricles. **(C)** Protein levels of RV collagen 1. **(D,E)** Results of fibrosis quantification on Masson’s trichrome stainings, expressed as percentage blue-stained area. **(F)** Representative examples of Masson’s trichrome fibrosis stainings. White bars represent 200 μm, and black bars represent 4 mm. **(G)** Representative examples of Sirius red fibrosis stainings. White bars represent 200 μm, and black bars represent 4 mm. * represents significantly different from control, and bar represents significantly different between ACS groups.

**FIGURE 6:**
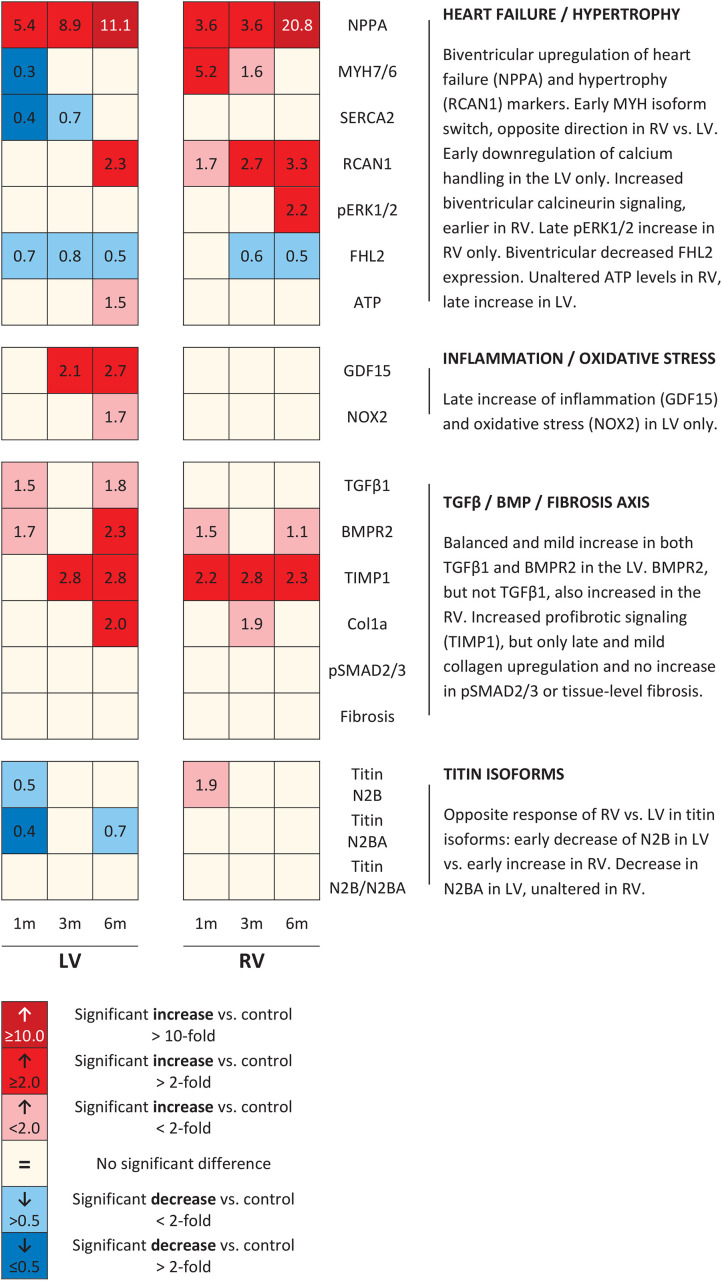
Schematic overview of molecular changes. Schematic representation of results of all molecular remodeling analyses, displayed in heatmap format. Rows represent separate analyses, and columns represent time points.

The expression of TGFβ 1, a potent inducer of fibrosis, remained unaltered in the RV, but increased in the LV at 1 month and 6 months ACS (Figures 4A,B). Interestingly, a similar pattern was observed for TGFβ’s counterpart BMPR2, a member of the TGFβ superfamily that plays a protective role in different forms of fibrosis (Bryant et al., 2015; Gilbane et al., 2015; Rol et al., 2018), that only slightly increased in the RV at 1 month and 6 months ACS, and increased in the LV at 1 month and 6 months ACS (Figures 4C,D). In the RV, but especially in the LV, TGFβ1 and BMPR2 expression showed a close correlation (Figures 4K,L). Growth/differentiation factor 15 (GDF15), another member of the TGFβ superfamily related to inflammation, remained unaltered in the RV of ACS rats, but increased in the LV at 3 and 6 months ACS, compared to control (Figures 4E,F). Tissue inhibitor of metalloproteinases 1 (TIMP1), a TGFβ target gene, increased in the RV at all time points, and in the LV at 3 and 6 months (Figures 4G,H). The indirect marker of oxidative stress NADPH oxidase 2 (NOX2) remained unaltered in the RV of ACS rats, and increased in the LV at 6 months ACS, compared to control (Figures 4I,J). Collagen 1a (Col1a) expression slightly increased at 3 months in the RV in ACS rats, and slightly increased in the LV at 6 months ACS, compared to control (Figures 5A,B). At protein level, there was no increase in collagen 1 in the RV of shunt animals, but rather a decrease (Figure 5C), and there was no increase in both ventricles of phospho-SMAD2/3 in ACS rats, compared to control (Figure 4M). Also histologically, there was no increased fibrosis in the RV nor in the LV of ACS rats, compared to control at any time point (Figures 5F,G). Qualitative assessment demonstrated no perivascular areas of fibrosis. A schematic overview of remodeling and fibrosis results is displayed in Figure 6.

## Discussion

The present study demonstrates that the ACS rat model of long-term volume load leads to end-stage RV failure after 6 months. At this point, clinical signs of end-stage RV failure had developed (pleural effusion, liver edema, ascites), along with cardiac hypertrophy and severely impaired RV contractility. Furthermore, gradual decreases of both RV relaxation and LV contractility had occurred, which were relatively mild in comparison to the decrease in RV contractility. Myocardial fibrosis was, however, not observed, nor was activation of pro-fibrotic pathways. Our data add to the growing evidence that, different from LV failure, fibrosis is not a hallmark of RV failure associated with chronic volume overload.

The steep decrease of RV contractility (Ees) at 6 months, in contrast with the more gradual and less severe decrease of LV function, indicates that the symptomatic heart failure in the current model is predominantly driven by RV failure. Also, the significant decrease of RV contractility at 6 months, in contrast with the more gradual impairment of RV relaxation (dP/dt_min_), implicates RV failure due to decreased contractility. This is in contrast with the pressure-loaded RV, in which clinical symptoms of RV failure have been associated with diastolic, more than systolic dysfunction (Borgdorff et al., 2015). These observations demonstrate that distinct RV loading conditions not only reveal specific patterns of remodeling, but also differential functional culprits in the transition from compensated to decompensated RV failure. The observed increase in TAPSE, a measure of longitudinal RV shortening that is very dependent of volume loading, may seem contradictory to the steeply decreased RV Ees, but this presumably reflects the severe degree of RV volume loading, rather than systolic RV function. In ACS rats, there was no substantial increase in RV peak pressures and no signs of pulmonary vascular remodeling could be demonstrated. In other words, in the absence of any signs of increased RV pressure load, this ACS model can be regarded as a model of isolated volume overload, by definition similar for the RV and LV. Therefore, this animal model provides fair comparability of RV and LV adaptation to similar volume loading. The findings in the current study, showing predominant RV failure instead of LV failure, indicate that the LV has the potential to adapt better to volume load than the RV.

A pivotal finding in this study is that no myocardial fibrosis could be demonstrated, despite the end-stage degree of RV failure. Also in terms of pro-fibrotic signaling in ACS rats, only minor and balanced changes were observed compared to controls. For example, in the RV, only an increase in TIMP1 was found, but no concurrent alterations in TGFβ1 expression, no inflammation in terms of GDF15 expression, and no signs of oxidative stress (NOX2). Subsequently, this resulted in barely altered collagen gene expression and unaltered levels of phosphorylated SMAD2/3. This all is in accordance with the absence of increased protein levels of collagen, or fibrosis by histological assessment. Also, in both ventricles, TGFβ1 and BMPR2 expression were balanced, illustrated by their linear correlation. This balance and the unaltered levels of phosphorylated SMAD2/3 are also in line with the absence of myocardial fibrosis, as BMPR2 is an antagonist in the TGFβ fibrotic pathway, preventing fibrosis (Goumans et al., 2008). At 6 months, some signals of inflammation, oxidative stress and collagen expression could be observed in the LV. These might reflect a reactive phenomenon considering the later stages at which these signals occurred, and considering that these signals did not result in protein-level or tissue-level increases in collagen deposition. Altogether, in the course toward end-stage RV failure, there were only mild and late, and mostly left-sided changes in pro-fibrotic signaling, which did not result in increased tissue fibrosis.

These findings challenge the dogma that myocardial fibrosis, either as cause or consequence, is associated with volume load-induced end-stage RV failure. As discussed earlier, previous studies in models of RV pressure load have demonstrated that targeting RV fibrosis does not improve ventricular function (Crnkovic et al., 2018) and that rats with symptomatic RV failure showed less RV fibrosis than those without clinical symptoms (Borgdorff et al., 2015). In line with these reports on pressure loaded RV, the present study demonstrates that also in volume loaded RV, there is no relation between myocardial fibrosis and RV function. There are, however, reports of fibrosis in the context of experimental RV volume loading, albeit in the presence of confounding factors. For example, one study reports fibrosis in a model of RV volume loading induced by creating pulmonary valve insufficiency in mice (Reddy et al., 2013). This study describes patchy subendocardial fibrosis, instead of diffuse interstitial fibrosis, and the use of myocardial stitches to induce valvular insufficiency may have triggered subendocardial hypo-perfusion and subsequent formation of fibrotic areas. Another study describes cardiac fibrosis in pigs with aortocaval shunt, but these pigs demonstrated increased RV pressures, inducing a mixed load instead of isolated volume load (Modesti et al., 2004).

The absence of myocardial fibrosis in the present animal model of isolated volume load-induced RV failure, and the consequential lack of a relation with ventricular function, may have relevant consequences for the interpretation of imaging-based studies, reported earlier. These studies demonstrate indirect markers of increased fibrosis in the RV of human subjects with rTOF, who are often subjected to chronic RV volume load due to pulmonary valve incompetence (Haggerty et al., 2017; Hanneman et al., 2017; Yim et al., 2017; Cochet et al., 2019). Now experimental studies increasingly disconnect RV fibrosis from function (Crnkovic et al., 2018), it seems more likely that the observed markers in humans depict processes that are secondary to remodeling or adverse loading conditions prior to surgical repair, rather than primary processes that are detrimental and are caused by volume loading. Even though it may seem hopeful that a potentially treatable process as fibrosis correlates with disease severity and cardiac function, it is not necessarily a suitable target of treatment, as has been suggested (Hanneman et al., 2017).

The present study demonstrates a certain degree of impaired RV relaxation, in terms of RV dP/dt_min_, that apparently has not been caused by increased deposition of collagens. Previous reports on volume-loaded LV remodeling showed that not interstitial stiffness, but sarcomeric stiffness may be responsible impaired LV relaxation (Hutchinson et al., 2015; Mohamed et al., 2016a,b). In cardiac muscle, titin’s isoforms N2B and N2BA are most important, of which N2B is stiffer, and N2BA more compliant (Lewinter and Granzier, 2014). In the present study, titin’s stiffer isoform N2B was increased at 1 month ACS in the RV, and the more compliant isoform N2BA remained unaltered. Even though the ratio was not significantly altered, the increase in N2B may have induced increased RV sarcomeric stiffness. This may be a compensatory mechanism for the decreasing myocardial stiffness due to volume load, which has been demonstrated previously (Chaturvedi et al., 2010). Interestingly, an opposite response of the LV was observed, with decreases of both isoforms. At 6 months ACS; however, only the more compliant isoform N2BA was decreased, suggesting that not only in the RV, but also in the LV, N2B expression is relatively more pronounced, compared to N2BA, suggesting increased sarcomeric stiffness.

Chronic volume loading further resulted in ventricle-specific molecular adaptation patterns. Both ventricles demonstrated a strong increase in NPPA expression, but the increase in NPPA expression was most abundant in the RV, in agreement with the most pronounced dysfunction of the RV, compared to the LV. Levels of ATP remained unaltered in the RV, and thus challenge the ‘engine out of fuel’ concept (Neubauer, 2007), as has also been done recently in the pressure-loaded RV (Koop et al., 2019). RV adaptation further comprises increased RCAN1 expression, MHC isoform switch and increased pERK1/2 protein expression, indicative of calcineurin activation and a stress-related hypertrophic response (Mercadier et al., 1981; Wilkins et al., 2004). ERK1/2 signaling has been identified as regulator between eccentric and concentric growth (Kehat et al., 2011), and since volume overload has been associated with dilating/eccentric growth, one may not expect an increase in myocardial pERK1/2 expression in the ACS-rats. However, a progressive increase in the RV cardiomyocyte cross-sectional area was observed in ACS rats, a measure of cardiomyocyte thickness. As increasing cardiomyocyte thickness is usually observed in concentric growth, this could depict a concentric, compensating mechanism to limit further dilatation at the 6 months time point. The LV, however, does not demonstrate a MHC isoform switch, nor were protein expression levels of pERK1/2 altered. The late increase of LV ATP levels potentially reflects a compensatory mechanism to maintain LV function. The LV further demonstrated decreased calcium handling (SERCA2a), which is in line with previous reports, whereas this was not observed in the RV (Mohamed et al., 2016b). As SERCA2a modulates excitation–contraction coupling (Frank et al., 2003), this may have contributed to the gradually decreasing LV systolic function observed in the present study. SERCA2a regulation has been posed as potential factor in RV failure in a research statement of the American Thoracic Society (Lahm et al., 2018), but the lack of altered SERCA2a in RV of ACS rats in the present study, despite clinical RV failure, suggests that SERCA2a is not involved in volume-loaded RV failure.

An interesting observation is the ubiquitous downregulation of FHL2 gene expression in ACS rats. FHL2 is known to inhibit phosphorylation of ERK1/2 and calcineurin activity, and therewith dampens cardiac hypertrophic responses (Tran et al., 2016). Thus, the decreased FHL2 expression may have resulted in the increase of pERK1/2 protein expression and RCAN1 gene expression in ACS rats. Furthermore, FHL2 is involved in sarcomere integrity, partly through interaction with actin and titin (Johannessen et al., 2006), and FHL2 mutations are associated with both dilated (Arimura et al., 2007) and hypertrophic (Friedrich et al., 2014) cardiomyopathy, and FHL2 is thus believed to contain cardioprotective properties. Prolonged downregulation of FHL2 expression therefore may have contributed to the gradual decrease of biventricular function.

A limitation of the present study is that longitudinal sections of the myocardium have been obtained, as displayed in Figures 5F,G, which do not include the RV insertion sites, or hinge points. These hinge points are clinically shown to be susceptible to focal fibrosis in patients with rTOF (Haggerty et al., 2017). However, even if there would have been some hinge point fibrosis, that we then have missed using longitudinal sections, these regions would be small and focal. Moreover, the overall pattern of little to no pro-fibrotic signaling has been derived from whole-ventricle tissue, including those regions. Therefore this limitation is considered to not come with a functionally relevant bias that could have altered conclusions. Another point to take into consideration when reading this manuscript is the high amount of statistical comparisons. Despite that the false discovery rate was controlled in the statistical methodology, the high amount of comparisons merits the readers’ critical appraisal of patterns, rather than individual *p*-values.

In conclusion, the present study demonstrates that in a rat model of long-term volume overload, clinical signs of RV failure occur at 6 months. At this point, RV contractility steeply decreased, while LV function remained relatively spared, in accordance with a more abundant increase of natriuretic peptide expression in the RV. However, despite the end-stage degree of RV failure, no myocardial fibrosis or pro-fibrotic signaling was observed. These findings indicate that myocardial fibrosis is not involved in the transition from compensated to decompensated RV dysfunction in this model of long-term volume loading.

## Data Availability Statement

The raw data supporting the conclusions of this article will be made available by the authors, without undue reservation.

## Ethics Statement

The animal study was reviewed and approved by the Dutch Central Ethical Committee for Animal Experiments and the Animal Care Committee of the University Medical Center Groningen.

## Author Contributions

QH and RMB conceived and planned the study. QH mainly performed the animal experiments. A-MK, GB, TL, and DF helped with the animal experiments. QH, KK, EM, and TL performed laboratory analyses. QH, KK, A-MK, GB, DF, RAB, M-JG, and RMB contributed to the interpretation of the results. QH drafted the manuscript. RAB, M-JG, and RMB supervised this study. All authors provided critical feedback, revised the manuscript, and approved with its final form.

## Conflict of Interest

The University Medical Center Groningen (UMCG) has received fees for consultancy activities of RMB for Actelion and Lilly outside the content of this manuscript. The UMCG has received research grants and/or fees from AstraZeneca, Abbott, Bristol-Myers Squibb, Novartis, Novo Nordisk, Roche, Trevena, and ThermoFisher GmbH for projects of RAB, outside the content of this manuscript. RAB is a minority shareholder of scPharmaceuticals, Inc. and received personal fees from MandalMed Inc., AstraZeneca, Novartis, Servier, and Vifor. The remaining authors declare that the research was conducted in the absence of any commercial or financial relationships that could be construed as a potential conflict of interest.
